# Solamargine Alleviates Proliferation and Metastasis of Cervical Cancer Cells by Blocking the CXCL3-Mediated Erk Signaling Pathway

**DOI:** 10.1155/2022/7634754

**Published:** 2022-10-29

**Authors:** Xiangdong Qu, Jirong Xie, Youyang Zhang, Zhimin Wang

**Affiliations:** ^1^Department of Gynecology, Taizhou Central Hospital (Taizhou University Hospital), Taizhou, China; ^2^Department of Obstetrics and Gynecology, Taizhou Central Hospital (Taizhou University Hospital), Taizhou, China; ^3^Department of Integrated Traditional Chinese and Western Medicine, Taizhou Central Hospital (Taizhou University Hospital), Taizhou, China

## Abstract

Solamargine has unique antitumor efficacy in a variety of cancers. The study is to explore the role of solamargine in cervical cancer. HeLa and SiHa cells were exposed to solamargine treatment at divergent concentrations (0, 5, 10, and 20 *μ*M). The antitumor role of solamargine in cervical cancer cells was determined by cell counting kit 8 (CCK-8), colony formation, scratch test, transwell assay, and western blot. The expression of mRNAs regulating the extracellular regulated protein kinases (Erk) pathway in solamargine-treated cells was detected by qRT-PCR. Rescue experiments were conducted to explore the effect of C-X-C motif chemokine ligand 3 (CXCL3). Following that, we inhibited Erk1/2 by PD98059 to investigate the interplay between CXCL3 and Erk pathway in solamargine-treated cells by measuring migration, invasion, and related matrix metalloproteinase (MMP) expressions. Solamargine inhibited the viability, proliferation, migration, and invasion of cervical cancer cells in a dose-dependent manner. The expression of p-Erk1/2 was downregulated by solamargine. CXCL3 overexpression abrogated the antitumor effect of solamargine on cervical cancer cells. The inhibition of the Erk signaling pathway restored the inhibiting role of solamargine which interfered with CXCL3 overexpression, in invasion, migration, and expressions of MMP-2 and MMP-9 in cervical cancer cells. Moreover, solamargine inhibited the growth of tumor *in vivo* xenograft model. Solamargine alleviated proliferation and metastasis of cervical cancer cells by blocking the CXCL3-mediated Erk signaling pathway.

## 1. Introduction

Cervical cancer is one of the malignant tumors of the female reproductive system [1]. According to statistics, the incidence of cervical cancer remains high with easy metastasis and recurrence, posing a serious threat to the health of women's lives [2]. It has been shown that the onset and development of cervical cancer is the result of a combination of genetic and environmental factors, and many aspects of its etiology are still unclear [3]. Therefore, the study of the pathogenesis of cervical cancer and its metastatic mechanism is urgent for its current treatment and prognosis.

In recent years, the multitarget, integrated regulation, and low side effect characteristics of traditional Chinese medicine have become a boom in the current research of tumor treatment [4, 5]. Chinese herb Long Kui (*Solanum nigrum* L.) contains various steroidal alkaloids that possess a wide range of bioactive effects such as antitumor and anti-inflammatory [6–8]. Solamargine extracted from the herb have been widely studied for its strong antitumor property in multiple cancers including breast, prostate, and lung cancers [9–11]. Presently, the molecular mechanism of this herbal monomer in cervical cancer has not been elucidated.

Extracellular-regulated protein kinases (Erk) pathway belongs to the mitogen-activated protein kinase (MAPK) family pathways, mediating important signals from extracellular stimuli to intracellular responses, and is closely associated with human malignant tumorigenesis [12, 13]. Numerous studies have demonstrated that the Erk pathway is active in cervical cancer cells contributing to tumor development, infiltration, and metastasis [14–17]. Besides, by inhibiting the Erk pathway in cervical tumor-bearing animals, the growth of tumors can be effectively curbed [18]. Therefore, the regulating function of the Erk pathway in cervical cancer has enraptured researchers exploring the possibility of natural compounds that have an inhibitory effect on this pathway. Recently, it has been reported that solamargine inhibits the phosphorylation of Erk1/2 in gastric cancer and exerts an anticancer effect [19]. However, there is a lack of reports on whether solamargine can modulate the Erk pathway in cervical cancer.

Chemokines are a group of small molecular proteins with a molecular weight of approximately 8–12 kDa that have the ability to induce targeted chemotaxis of responding cells in vicinity [20, 21]. Increasingly, chemokine family members and their coreceptors have been shown to play a role in the proliferation, apoptosis, invasion, or angiogenesis of a variety of cancer cells. C-X-C motif chemokine ligand 3 (CXCL3), as a member of the chemokine family, has been found to be closely associated with tumor formation via the Erk pathway [22].

In this study, we investigated the potential antitumor effect of solamargine on cervical cancer and revealed a finding that solamargine could suppress the growth and metastasis of cervical cancer cells mediated by the CXCL3-regulated Erk signaling pathway, which provided a potential therapeutic strategy for cervical cancer.

## 2. Methods

### 2.1. Cell Line and Culture

Human normal cervical cell line Ect1/E6E7 (CRL-2614) and cervical cancer cell lines HeLa (CCL-2) and SiHa (HTB-35) provided by American Type Culture Collection (ATCC, USA) were used as research objects. All cells were cultured in Eagle's Minimum Essential Medium (30–2003, ATCC, USA) supplementing with 10% fetal bovine serum (FBS, 30–2020, ATCC, USA). The incubator environment for cells was set at 37°C and 5% CO_2_.

### 2.2. Solamargine Treatment

After subculturing, the cells were harvested for solamargine treatment as previously described [23]. In brief, solamargine powder (BP1320, purity >98%) obtained from Chengdu Biopurify Phytochemicals Ltd. (Sichuan China) was dissolved in dimethyl sulfoxide (DMSO, D274279, Aladdin, China) for preparing 0, 5, 10, and 20 *μ*M solamargine solution. Then, the cells suspended in a 96-well plate (2 × 10^3^ cells/well) were treated with different concentrations of solamargine for 24 hours (h) at 37°C. Following treatment, the cell counting kit-8 (CCK-8) assay was used to detect cell viability.

### 2.3. Transient Transfection Assay

To explore the role of CXCL3 in the cells under solamargine treatment (10 *μ*M), we transfected plasmid targeting CXCL3 (pEZ-CXCL3, GenePharma, China) into HeLa and SiHa cells by Lipofectamine 2000 (11668019, Invitrogen, USA) according to the protocols. Empty-plasmid-transfected cells served as the negative control (NC). Quantitative real-time polymerase chain reaction (qRT-PCR) was then carried out for evaluating transfection effect.

### 2.4. Extracellular-Regulated Protein Kinase (Erk) Inhibition

After cell transfection, the cells were treated with Erk inhibitor PD98059 (20 *μ*M, Calbiochem, San Diego, CA, USA) for 24 h at 37°C with 5% CO_2_ as previously described [15].

### 2.5. CCK-8 Assay

For evaluating the viability of solamargine-treated cells, we employed CCK-8 kit assay (CK04, Dojindo, Japan). The cells were resuspended in the 96-well plate and reacted with CCK-8 solution (10 *μ*L/well) for 4 h at 37°C. After that, a microplate reader (800TS, BioTek, USA) was used to detect the absorbance at a wavelength of 450 nm. Ect1/E6E7 cells treated with solamargine was used for the control.

### 2.6. Colony Formation Assay

After solamargine treatment (0, 5, or 10 *μ*M), colony formation assay was performed in HeLa and SiHa cells transfected with or without CXCL3-overexpressing plasmid for assessing proliferative capacity. The cells were collected and resuspended in a 6-well plate at a density of 5 × 10^2^ per well. The plate was then maintained in an incubator (37°C, 5% CO_2_) for 2 weeks. Subsequently, the proliferated cells were treated with 4% paraformaldehyde (P1110, Solarbio, China) for 30 minutes (min) followed by crystal violet dying (C196471, Aladdin, China) at room temperature. The images of stained colonies were captured by a microscope (CX23, Olympus, Japan).

### 2.7. Scratch Test

For measuring the migratory capacity of HeLa and SiHa cells in divergent processes, scratch test was carried out. The specified cells incubated in a 6-well plate (1 × 10^6^ cells/well) were assigned to a scratch in a straight line in each well using a pipette tip. After removing debris by phosphate-buffered saline (PBS, C0221 A, Beyotime, China), the cells were subjected to wound healing for 24 h. The images of migrated cells at 0 h and 24 h were photographed using the microscope (×100 magnification).

### 2.8. Transwell Assay

Transwell insert (354480, BD, USA) was employed to detect cell invasion. In short, the cells (2 × 10^5^) processed as required were resuspended with Eagle's Minimum Essential Medium and added into the upper chamber of the insert. Meanwhile, the medium containing 10% FBS was injected into the lower chamber as a chemoattractant. The insert was kept at 37°C for 24 h before being swabbed noninvaded cells adhering on the upper surface. Subsequently, the invaded cells were subject to paraformaldehyde and crystal violet treatments as similarly described in the colony formation assay. The results were observed under the microscope (×250 magnification).

### 2.9. RNA Extraction and qRT-PCR

To determine mRNA expressions, total RNA was extracted from HeLa and SiHa cells by homogenization in TRIzol reagent (T9424, Sigma-Aldrich, USA). Then, the extracts were treated with RT-qPCR kit (A-B4106 C, Themo Fisher, USA) according to the operating instructions. Following the reverse transcription, StepOne Real-Time PCR System (4376357, Thermo Fisher, USA) was conducted for qRT-PCR. Relative gene expressions were analyzed using the 2^−ΔΔCt^ method [24], with normalization of glyceraldehyde-3-phosphate dehydrogenase (GAPDH). Triplicate independent experiments were required in all samples. Synthesized primers used as follows:

PDZ binding kinase (PBK), forward: 5′-CCAAACATTGTTGGTTATCGTGC-3'; reverse: 5′-GGCTGGCTTTATATCGTTCTTCT-3′. Receptor for activated C kinase 1 (RACK1), forward: 5′-ACCATCATCATGTGGAAACTGAC-3'; reverse: 5′-GTGCCCGTTGTGAGATCCC-3′. Thyroid hormone receptor interactor 4 (TRIP4), forward: 5′-GAGAGTGCTGAAGAGATACGAGA-3'; reverse: 5′-AGATGGTCGCCTGATTTCTGC -3′. Semaphorin 3C (SEMA3C), forward: 5′-TTTGCGTGTTGGTTGGAGTAT-3'; reverse: 5′-TCCTGTAGTCTAAAGGATGGTGG-3′. CXCL3, forward: 5′-CGCCCAAACCGAAGTCATAG-3'; reverse: 5′-GCTCCCCTTGTTCAGTATCTTTT-3′. GAPDH, forward: 5′-ACAACTTTGGTATCGTGGAAGG-3'; reverse: 5′-GCCATCACGCCACAGTTTC-3′.

### 2.10. Western Blot

RIPA lysis buffer (E-BC-R327, Elabscience, China) was utilized to lyse total protein from HeLa and SiHa cells undergoing indicated treatments. Then, the lysates were assigned to protein quantification by BCA protein assay kit (P0010S, Beyotime, China). Equal amounts of protein were loaded onto PVDF membranes (AR0136-02, Boster, China) and blocked with 5% skimmed milk (P0216, Beyotime, China) as previously described [19]. Thereafter, the membranes were incubated with primary antibodies at 4°C overnight and then with secondary antibodies for 2 h at room temperature. Immunoreactive blots were determined by ECL Western Blotting Substrate (32209, Thermo Fisher, USA) and quantified using iBright Imaging System (CL750, Invitrogen, USA). The relative protein level of genes was normalized to GAPDH. All antibodies purchased from Abcam (UK) were anti-MMP-2 (ab92536, 1/1000, 74 kDa), anti-MMP-9 (ab38898, 1/1000, 92 kDa), anti-p-Erk1/2 (ab223500, 1/400, 44, 42 kDa), anti-Erk1/2 (ab184699, 1/10000, 44, 42 kDa), anti-CXCL3 (ab220431, 1/1000, 11 kDa), anti-GAPDH (ab8245, 1/500, 36 kDa), anti-mouse IgG (ab205719, 1/2000) and anti-rabbit IgG (ab97051, 1/2000).

### 2.11. Nude Mouse Tumor Cell Xenograft Analysis

A total of 12 female specific pathogen-free BALB/*c* nude mice (6–8 weeks old, 18–20 g) were purchased and housed under 21 ± 2 °C, 50–60% humidity with a 12 h light/dark cycle and with food and water provided. Mice were divided to the control and solamargine groups (*n* = 6/group), and HeLa cells (5 × 10^5^ cells) were injected subcutaneously into nude mice of each group. After 10 days, the solamargine group was treated with 10 mg/kg solamargine once daily by intragastric administration, while the control group was administered with PBS. Tumor volume was measured every 3 days, and the volume was calculated by the formula: V = *l*/2 x (length* × *width^2^). Mice were sacrificed for the collection of tumor samples on day 21.

### 2.12. Statistical Analysis

GraphPad Prism (vision 8.0, GraphPad Software, USA) was leveraged to analyze statistics, and measurement data were expressed as mean ± standard deviation. One-way analysis of variance was used for comparison of differences between groups, with differences considered statistically significant at *p* < 0.05.

## 3. Results

### 3.1. Solamargine Inhibited the Viability and Proliferative Capacity of Cervical Cancer Cells

**Figure 1:**
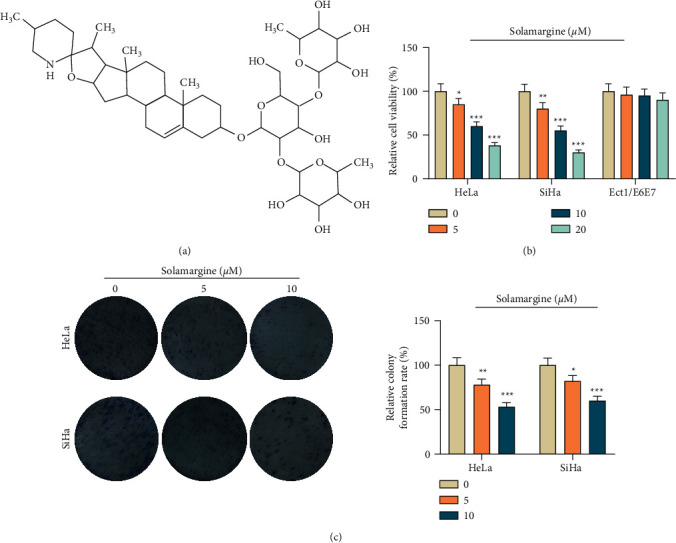
The effect of solamargine on the viability and proliferation of cervical cancer cells. (a) The chemical structure of solamargine. (b) CCK-8 was used to evaluate the viability of cervical cancer cells under 0, 5, 10, and 20 *μ*M solamargine treatment. (c) Colony formation assay was used to detect the proliferation of cervical cancer cells under 0, 5, and 10 *μ*M solamargine treatment. ^*∗*^*p* < 0.05, ^*∗∗*^*p* < 0.01, and ^*∗∗∗*^*p* < 0.001 vs. 0 *μ*M. CCK-8, cell counting kit 8.

The image displayed in Figure 1(a) was the chemical structure of solamargine. In the CCK-8 analysis, the viability of both HeLa and SiHa cells, which were treated with solamargine (0, 5, 10 and 20 *μ*M) for 24 h, was strikingly restrained in a dose-dependent manner, whereas solamargine had no significant effect on the viability of Ect1/E6E7 cells (Figure 1(b), *p* < 0.05). As solamargine inhibited cell viability too strongly at a concentration of 20 *µ*M, 5 and 10 *μ*M of the drug were selected for subsequent investigations. The results of colony formation assay indicated that the proliferation of tumor cells was suppressed by solamargine in a concentration-dependent way (Figure 1(c), *p* < 0.05).

### 3.2. Solamargine Dampened the Migration and Invasion of Cervical Cancer Cells

**Figure 2:**
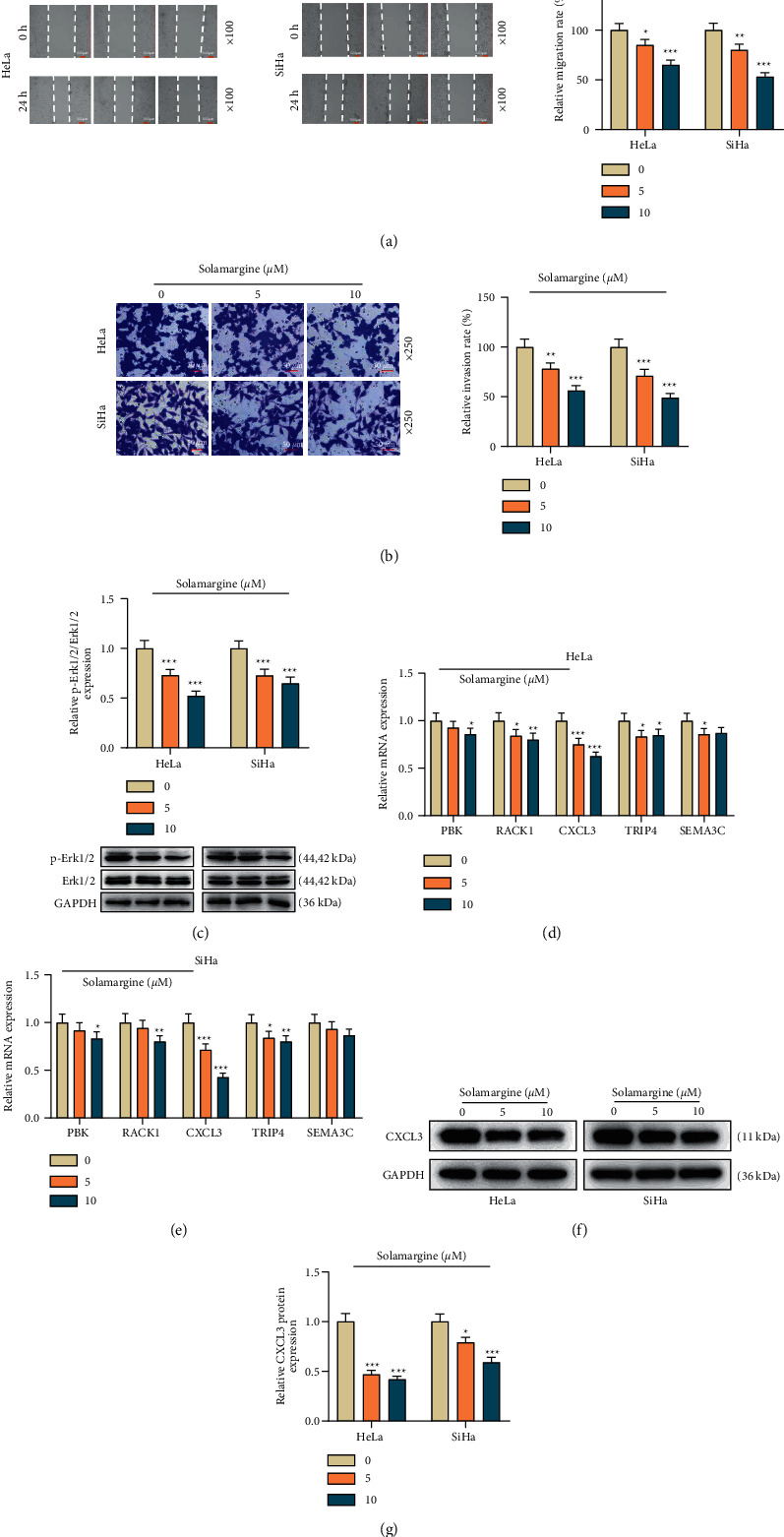
The effect of solamargine on the metastasis of cervical cancer cells and its potential mechanism related to the Erk pathway. (a) Scratch test was performed to assess the migration of tumor cells under 0, 5, and 10 *μ*M solamargine treatment after 24 h (×100 magnification, scale bar = 100 *μ*m). (b) Transwell assay was used to measure the invasion of solamargine-treated cells (×250 magnification, scale bar = 50 *μ*m). (c) Following exposure to solamargine, western blot was conducted to detect p-Erk1/2 and Erk1/2 protein levels. GAPDH served as the loading control. (d, e) qRT-PCR was performed to detect the expression of PBK, RACK1, CXCL3, TRIP4, and SEMA3C in solamargine-treated cells. GAPDH served as the internal control. (f, g) Western blot was conducted to detect the protein expression of CXCL3 in solamargine-treated cells. ^*∗*^*p* < 0.05, ^*∗∗*^*p* < 0.01, and ^*∗∗∗*^*p* < 0.001 vs. 0 *μ*M. h, hours; Erk, extracellular regulated protein kinases; p, phosphorylated; qRT-PCR, quantitative real-time polymerase chain reaction; GAPDH, glyceraldehyde-3-phosphate dehydrogenase; PBK, PDZ binding kinase; RACK1, receptor for activated C kinase 1; CXCL3, C-X-C motif chemokine ligand 3; TRIP4, thyroid hormone receptor interactor 4; SEMA3C, semaphorin 3C.

In the migration assay, a sluggish propensity of wound healing was observed in the solamargine-treated cells compared to the untreated ones (Figure 2(a), *p* < 0.05). Similarly, the results of transwell assay showed that the invasive capacity of 5 and 10 *μ*M solamargine-treated cells was declined compared to that of the untreated group (Figure 2(b), *p* < 0.01).

### 3.3. Solamargine Played a Suppressing Role in the Erk Signaling Pathway

Given that previous studies have confirmed that the Erk signaling pathway plays an important role in the progression of cervical cancer, we determined the level of pivotal proteins along this transmission in solamargine-treated cells by western blot. As shown in Figure 2(c), (p)-Erk1/2 protein level was remarkably downregulated by solamargine compared to the control group (*p* < 0.001).

### 3.4. CXCL3 Overexpression Abrogated the Antitumor Effect of Solamargine on Cervical Cancer Cells

**Figure 3:**
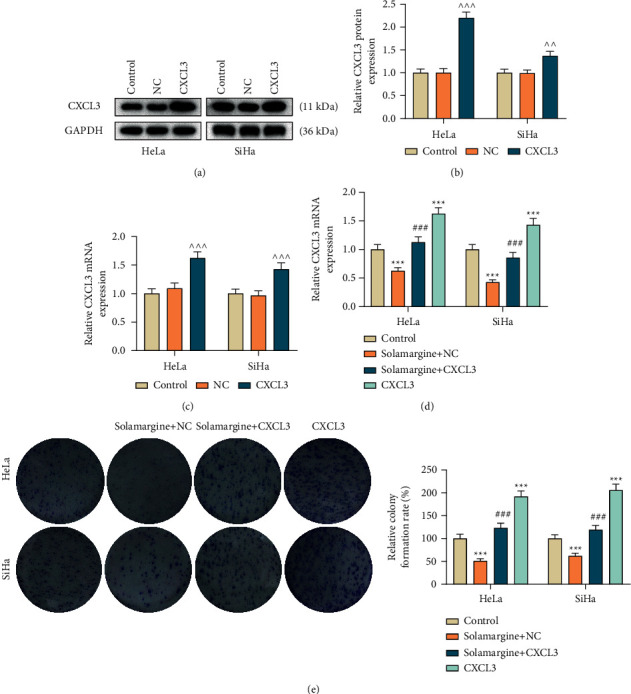
Solamargine regulated CXCL3 expression which played a role in the proliferation of cervical cancer cells. (a–c) The expression of CXCL3 in cervical cancer cells transfected with plasmid targeting CXCL3 was verified by qRT-PCR and western blot. GAPDH served as the internal control. (d) Following exposure to 10 *μ*M solamargine, qRT-PCR was used to detect the expression of CXCL3 in the transfected cells. GAPDH served as the internal control. (e) After CXCL3 overexpression, the proliferative capacity of solamargine-treated cells was determined by colony formation assay. ^∧∧∧^*p* < 0.001 vs. NC; ^*∗∗∗*^*p* < 0.001 vs. control; ^###^*p* < 0.001 vs. solamargine + NC. qRT-PCR, quantitative real-time polymerase chain reaction; GAPDH, glyceraldehyde-3-phosphate dehydrogenase; CXCL3, C-X-C motif chemokine ligand 3; NC, negative control.

**Figure 4:**
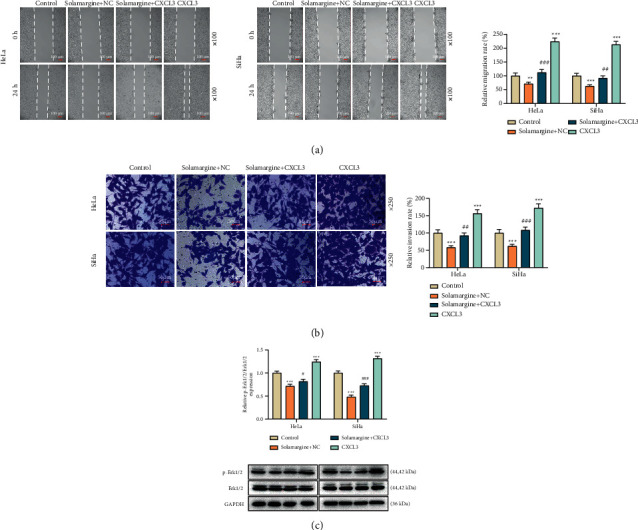
Solamargine regulated CXCL3 expression which played a role in the metastasis of cervical cancer cells and the Erk pathway. (a) Wound healing assay was performed to evaluate the migration of cervical cancer cells after CXCL3 overexpression transfection and 24 h of 10 *μ*M solamargine treatment (×100 magnification, scale bar = 100 *μ*m). (b) Following cell migration detection, transwell assay was used to measure cell invasion (×250 magnification, scale bar = 50 *μ*m). (c) After 10 *μ*M solamargine treatment, the protein level of p-Erk1/2 and Erk1/2 in the transfected cells was determined by western blot. GAPDH served as the loading control. ^*∗∗*^*p* < 0.01 and ^*∗∗∗*^*p* < 0.001 vs. control; ^#^*p* < 0.05, ^##^*p* < 0.01, and ^###^*p* < 0.001 vs. solamargine + NC. GAPDH, glyceraldehyde-3-phosphate dehydrogenase; CXCL3, C-X-C motif chemokine ligand 3; Erk, extracellular regulated protein kinases; p, phosphorylated; h, hours; NC, negative control.

In order to investigate whether solamargine regulates the Erk signaling pathway by modulating target mRNAs, we screened out genes that regulate this pathway in cervical cancer including PBK, RACK1, CXCL3, TRIP4, and SEMA3C, which have been reported in the literature in recent years [25–29]. By qRT-PCR, we found that the expression of all of the above genes was downregulated in HeLa and SiHa cells treated by solamargine, with CXCL3 being the most significantly regulated by this monomer (Figures 2(d) and 2(e), *p* < 0.05), so we chose to do further studies on CXCL3 by rescue experiments. In addition, the protein expression of CXCL3 in solamargine-treated HeLa and SiHa cells was also detected, and the result showed that solamargine inhibited the protein expression of CXCL3 (Figures 2(f) and 2(g), *p* < 0.05). Before that, we conducted cell transfection to obtain CXCL3 overexpression in HeLa and SiHa cells, and the results of western blot and qRT-PCR validated that the protein and mRNA expression of CXCL3 was elevated by CXCL3 overexpression (Figures 3(a) and 3(c), *p* < 0.01). After solamargine incubation, the promoting effect of CXCL3-overexpressing plasmid on CXCL3 expression was significantly overturned (Figure 3(d), *p* < 0.001). In rescue experiments, the upregulation of CXCL3 bolstered the malignant phenotype of cervical cancer cells in proliferation (Figure 3(e), *p* < 0.001), migration (Figure 4(a), *p* < 0.001), and invasion (Figure 4(b), *p* < 0.001) compared to its control group, which also starkly reversed the antitumor effect of solamargine on cell proliferation (Figure 3(e), *p* < 0.001), migration (Figure 4(a), *p* < 0.01), and invasion (Figure 4(b), *p* < 0.01). Besides, the results of western blot demonstrated that the CXCL3 overexpression promoted the expression of p-Erk1/2 compared to the control group (Figure 4(c), *p* < 0.001), but it partially reversed the suppressing role of solamargine in the p-Erk1/2 expression compared to the group treated with solamargine alone (Figure 4(c), *p* < 0.001).

### 3.5. The Erk Signaling Pathway Regulated by CXCL3 Participated in the Antitumor Mechanism of Solamargine in Cervical Cancer

**Figure 5:**
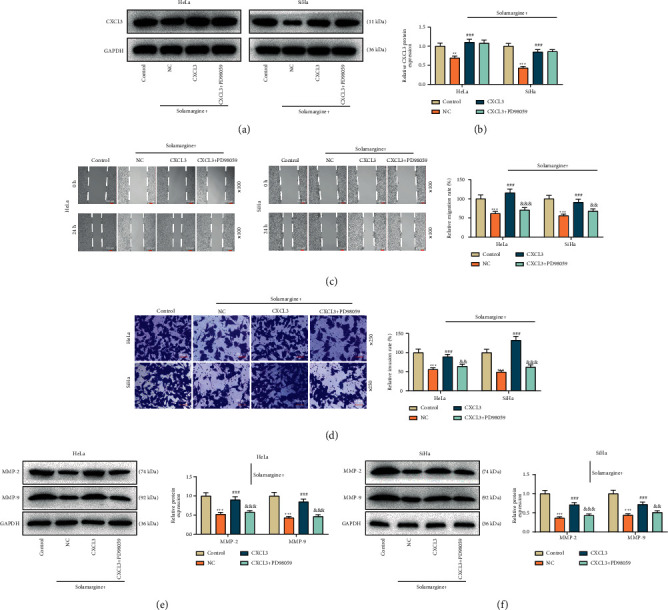
CXCL3 expression regulated the Erk pathway to influence the metastasis of solamargine-cervical cancer cells. (a, b) Solamargine-treated cells with CXCL3 overexpression in the absence or presence of PD98059 (specific Erk1/2 inhibitor) for 24 h; the protein expression of CXCL3 was detected by western blot. (c) The cervical cancer cells were treated as above and measured the cell migration by scratch test (×100 magnification, scale bar = 100 *μ*m). (d) The cervical cancer cells were treated as above and measured invasive capacity by transwell assay (×250 magnification, scale bar = 50 *μ*m). (e, f) Western blot was performed to measure MMP-2 and MMP-9 protein levels in solamargine-treated cells after the upregulation of CXCL3 and the inhibition of the Erk pathway. GAPDH served as the loading control. ^*∗∗∗*^*p* < 0.001 vs. control; ^###^*p* < 0.001 vs. solamargine + NC; and ^&^*p* < 0.01 and ^&&^*p* < 0.001 vs. solamargine + CXCL3. h, hours; MMP-2, matrix metalloproteinase 2; MMP-9, matrix metalloproteinase 9; CXCL3, C-X-C motif chemokine ligand 3; NC, negative control.

Based on available findings that the involvement of CXCL3 depletion in the anticancer mechanism of solamargine might be entwined with the depression of the Erk signaling pathway, we applied PD98059 to block Erk1/2 in cervical cancer cells in a follow-up study and obtained more intuitive results. As revealed in Figures 5(a) and 5(b), the protein expression of CXCL3 was inhibited by solamargine, while CXCL3 overexpression partially reversed the inhibitory effect of solamargine (Figures 5(a) and 5(b), *p* < 0.01), and there was no significant difference in CXCL3 protein expression between the solamargine + CXCL3 group and solamargine + CXCL3+PD98059 group (Figures 5(a) and 5(b)). PD98059 visibly restored the inhibiting effect of solamargine on cell migration which were attenuated by CXCL3 overexpression (Figure 5(c), *p* < 0.001). Additionally, the similar result was observed in the sequent cell invasion determination (Figure 5(d), *p* < 0.01). Furthermore, we detected markers associated with tumor metastasis via western blot and found that solamargine downregulated protein levels of MMP2 and MMP9 in cervical cancer cells (Figures 5(e) and 5(f), *p* < 0.001), which were partially abrogated with the addition of CXCL3 overexpression (Figures 5(e) and 5(f), *p* < 0.001), whereas this effect of solamargine in CXCL3-overexpressing tumor cells was restored after Erk1/2 blocking (Figures 5(e) and 5(f), *p* < 0.01).

### 3.6. Solamargine Inhibited the Growth of Tumor in *In Vivo* Xenograft Model

**Figure 6:**
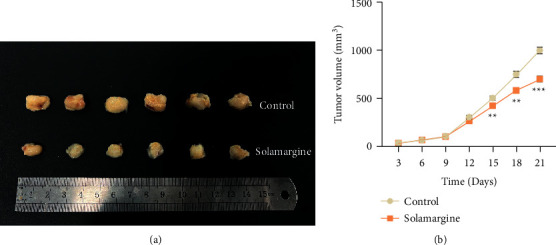
The effect of solamargine treatment in the xenograft mice model. (a) Mice were sacrificed, and the tumors were collected. (b) The tumor volume was measured in the control and solamargine group. ^*∗∗*^*p* < 0.01 and ^*∗∗∗*^*p* < 0.001 vs. control.

To further evaluate the role of solamargine in cervical cancer, the effect of solamargine on the tumor growth was investigated in a tumor xenograft model. As shown in Figures 6(a) and 6(b), the tumor volume in the solamargine group was significantly reduced compared with the control group. This suggested that solamargine exhibited an antitumor effect of cervical cancer *in vivo*.

## 4. Discussion

Chinese herbs have been shown to play an important role in the treatment of cancers so that they have become a popular topic in anticancer research [30]. Solamargine as an extract of *Solanum nigrum* Linn has antitumor activity and is specific in its efficacy in the treatment of many types of cancer. In the study of the metastasis mechanism of liver cancer, solamargine has been shown to significantly weaken the epithelial mesenchymal transition (EMT) of hepatocellular carcinoma cells, thereby acting as an inhibitor of tumor invasion and metastasis [23]. In addition, Xie et al. showed that the migration and invasion ability of pancreatic cancer cells were reduced and that the number of apoptotic cells was increased after induction by solamargine [31]. Current breakthroughs in cellular epigenetics have led to molecularly new insights into malignant tumorigenesis and progression [32]. Although clinical advances are being made in the diagnosis and treatment of cervical cancer, lymphatic metastases and poor prognosis that occur in its advanced stage remain problematic challenges [2, 33]. According to the above findings, in our previous study to probe into the potential effects of solamargine on cervical cancer, we found that solamargine had no significant effect on the viability of normal cells, but had an effective inhibitory effect on the viability, proliferation, migration, and invasion of cervical cancer cells, suggesting a possible anticancer mechanism of solamargine in the proliferation and metastasis of cervical cancer.

In cervical cancer research, the activation of the Erk signaling pathway is closely linked to the metastasis of cancer cells and is considered to be a therapeutic direction for cancer [15]. Erk includes Erk1 and Erk2, which are phosphorylated in response to cytokines, and the stimulated Erk1/2 is transferred to the nucleus to regulate related genes, thus engaging in various biological responses such as cell proliferation, invasion, and carcinogenesis [34]. Chen et al. found that the expression of p-Erk1 and p-Erk2 increased with the progression of cervical cancer and was identified as a hallmark of cancer differentiation [35]. Interestingly, our results of the p-Erk1/2 protein level in solamargine-treated cervical cancer cells were consistent with those of the former, which prompted us to presume that perhaps solamargine could modulate the Erk signaling pathway in cervical cancer.

Mechanically, to explore whether solamargine regulates the Erk pathway by targeting a specific mRNA, we selected genes reported in recent years to regulate the Erk pathway in cervical cancer, including PBK, RACK1, CXCL3, TRIP4, and SEMA3C, for study [25–29]. QRT-PCR demonstrated that CXCL3 was most significantly restricted by solamargine. CXCL3 belongs to the chemokine family and is primarily responsible for controlling the migration and adhesion of monocytes as well as affecting target cells through interaction with CXCR2 receptor on the cell surface [36]. It has been shown that CXCL3 interacts with CXCR2 to promote proliferation, metastasis, and invasion of endothelial cells, ultimately mediating angiogenesis [37]. The study by See et al. found that the CXCL3 expression was significantly upregulated in breast cancer tissues and supported it as a potential marker for evaluating breast cancer metastasis [38]. In our subsequent experiments, we found that the expression of CXCL3 in cervical cancer cells positively correlates with cell proliferation, migration, invasion, and the expression of p-Erk1/2. However, this promoting effect of CXCL3 was starkly reversed by solamargine, suggesting that CXCL3 may be the key to the inhibitory effect that solamargine could have on the Erk pathway. A study has shown that CXCL3 overexpression significantly promoted the migration of prostate cancer cells and increased the expression of the tumor-associated Erk in prostate cancer cells [22]. Qi et al. indicated that the CXCL3-induced MAPK/Erk pathway boosted the malignant phenotype of uterine cervical cancer cells [27]. In this study, we furthermore confirmed that solamargine exerted its anticancer effects by inhibiting CXCL3 to block phosphorylation of Erk1/2 by applying the Erk1/2 inhibitor PD98059 in wound healing and transwell assays. MMPs are protein hydrolases necessary for the degradation of the intercellular matrix during cell invasion [39]. Evidence from oncology studies suggests that activation of the MAPK/Erk signaling pathway could elevate the expression of MMP-2 and MMP-9 in different tumor cells, thereby enhancing their proliferation and invasion and metastasis [40, 41]. Intriguingly, we found that the effect of solamargine on MMP-2 and MMP-9 expressions in cervical cancer cells were similar to the findings of Sani et al. [42].

## 5. Conclusion

Collectively, our present study suggests that solamargine alleviated proliferation, migration, and invasion of cervical cancer cells by blocking the CXCL3-mediated Erk signaling pathway, and solamargine inhibited the tumor growth of mice *in vivo*. The findings obtained through *in vitro* and *in vivo* experiments may provide theoretical support for the potential application of solamargine in the treatment of cervical cancer.

## Data Availability

The analyzed data sets generated during the study are available from the corresponding author on reasonable request.
